# Fur Is a Repressor of Biofilm Formation in *Yersinia pestis*

**DOI:** 10.1371/journal.pone.0052392

**Published:** 2012-12-21

**Authors:** Fengjun Sun, He Gao, Yiquan Zhang, Li Wang, Nan Fang, Yafang Tan, Zhaobiao Guo, Peiyuan Xia, Dongsheng Zhou, Ruifu Yang

**Affiliations:** 1 State Key Laboratory of Pathogen and Biosecurity, Beijing Institute of Microbiology and Epidemiology, Beijing, China; 2 Department of Pharmacy, Southwest Hospital, the Third Military Medical University, Chongqing, China; 3 State Key Laboratory for Infectious Disease Prevention and Control, National Institute for Communicable Disease Control and Prevention, Chinese Centre for Disease Control and Prevention, Beijing, China; Tulane University, United States of America

## Abstract

**Background:**

*Yersinia pestis* synthesizes the attached biofilms in the flea proventriculus, which is important for the transmission of this pathogen by fleas. The *hmsHFRS* operons is responsible for the synthesis of exopolysaccharide (the major component of biofilm matrix), which is activated by the signaling molecule 3′, 5′-cyclic diguanylic acid (c-di-GMP) synthesized by the only two diguanylate cyclases HmsT, and YPO0449 (located in a putative operonYPO0450-0448).

**Methodology/Principal Findings:**

The phenotypic assays indicated that the transcriptional regulator Fur inhibited the *Y. pestis* biofilm production *in vitro* and on nematode. Two distinct Fur box-like sequences were predicted within the promoter-proximal region of *hmsT*, suggesting that *hmsT* might be a direct Fur target. The subsequent primer extension, LacZ fusion, electrophoretic mobility shift, and DNase I footprinting assays disclosed that Fur specifically bound to the *hmsT* promoter-proximal region for repressing the *hmsT* transcription. In contrast, Fur had no regulatory effect on *hmsHFRS* and YPO0450-0448 at the transcriptional level. The detection of intracellular c-di-GMP levels revealed that Fur inhibited the c-di-GMP production.

**Conclusions/Significance:**

*Y. pestis* Fur inhibits the c-di-GMP production through directly repressing the transcription of *hmsT*, and thus it acts as a repressor of biofilm formation. Since the relevant genetic contents for *fur*, *hmsT*, *hmsHFRS*, and YPO0450-0448 are extremely conserved between *Y. pestis* and typical *Y. pseudotuberculosis*, the above regulatory mechanisms can be applied to *Y. pseudotuberculosis*.

## Introduction

*Y. pestis* is highly virulent to mammalians including humans, and causes systemic and fatal infections mainly manifested as bubonic, septicemic, and pneumonic plague. *Y. pestis* is primarily transmitted via the bite of an infected flea. *Y. pestis* synthesizes the attached biofilms in the flea proventriculus, making the blockage of fleas [1], [2]. The blockage of fleas makes them feel hungry and repeatedly attempt to feed, and thus, the plague bacilli will be pumped into the host body during the futile feeding attempts, promoting the bacterial transmission between mammalian reservoirs [1], [2].

The *Yersinia* biofilms are a population of bacterial colonies embedded in the self-synthesized extracellular matrix, and the matrix is primarily composed of exopolysaccharide that is the homopolymer of *N*-acetyl-*D*-glucosamine [1]. The *hmsHFRS* operon is responsible for the synthesis and translocation of biofilm exopolysaccharide across the cell envelope, and all the four genes in this operon are required for the biofilm formation and for the flea blockage [1], [3].

The signaling molecule 3′, 5′-cyclic diguanylic acid (c-di-GMP) is a central positive activator of the enzymes catalyzing the production of biofilm exopolysaccharide [4]. HmsT [5], [6] and YPO0449 (y3730 in KIM) [7], [8] are the only two diguanylate cyclase enzymes in *Y. pestis* to synthesize c-di-GMP, and both of them stimulate the *Yersinia* biofilm formation. The predominant effect of HmsT was on the *in vitro* biofilm formation, while the role of YPO0449 in the biofilm production is much greater in the flea than *in vitro* [7].

The Rcs phosphorelay system negatively controls *Yersinia* biofilm production in both nematode and flea models [9]. The Rcs system is composed of the sensor kinase RcsC, the phosphotransfer RcsD, and the cytoplasmic response regulator RcsB [10]. RcsC and RcsD transfers phosphate to RcsB, and the phosphorylated RcsB (RcsB-P) binds to some of its target promoters to mediate the gene regulation, whereas a complex of RcsB-P and its accessory protein RcsA is required for the regulation of other target genes [10]. The RcsAB box sequence TAAGAAT-ATTCTTA is a 14 bp inverted repeat [11].

The ferric uptake regulator (Fur) is a predominant iron-regulating system in bacteria [12]. Fur directly controls not only almost of the iron assimilation functions but a variety of genes involved in various non-iron functions, and thus, this regulator governs a complex regulatory cascade in *Y. pestis* [13], [14]. Two consensus constructs, a 19 bp box and a position-specific scoring matrix (PSSM), have been built to represent the conserved *cis*-acting signals recognized by Fur [13]. The Fur box sequence AATGATAATNATTATCATT is a 9-1-9 inverted repeat.

During the general maintenance of *Y. pestis* on the agar media, we found that the *fur* mutant exhibited a much more rugose and dry colony morphology relative to its parent strain, which promoted us to hypothesize the Fur-mediated repression of exopolysaccharide synthesis and biofilm production in *Y. pestis* (see below for details). In the present work, the detection of biofilms verified that Fur inhibited the *Y. pestis* biofilm production *in vitro* and on nematode. The subsequent gene regulation experiments disclosed that Fur specifically bound to the promoter-proximal region of *hmsT* for repressing the *hmsT* transcription, and yet, it had no regulatory effect on *hmsHFRS* and YPO0450-0448. In addition, the detection of intracellular levels of c-di-GMP revealed that Fur inhibited the c-di-GMP production. Therefore, *Y. pestis* Fur inhibited the c-di-GMP production through directly repressing the transcription of *hmsT*, and thus, it acted as a repressor of biofilm formation.

## Materials and Methods

### Bacterial Strains and Growth

The wild-type (WT) *Y. pestis* biovar *Microtus* strain 201 is avirulent to humans but highly lethal to mice [15]. The entire coding region of *fur* or the base pairs 146 to 468 of *hmsS* was replaced by the kanamycin resistance cassette by using the one-step inactivation method based on the lambda phage recombination system, to generate the *fur* or *hmsS* null mutant (designated as *Δfur* or *ΔhmsS*, respectively) of *Y. pestis*, as described previously [14]. All the primers used in this study were listed in Table 1. Given the pervious observation that the deletion of *hmsS* lead to a biofilm-defective phenotype in *Y. pestis* [16], *ΔhmsS* was used as a reference biofilm-defective strain in this work.

**Table 1:** Primer used in this study.

Target gene	Primers (5′-3′; F/R)
Mutant construction	
*fur*	CAGCCTTAATTTGAATCGATTGTAACAGGACTGAATCCGCTGTAACGCACTGAGAAGC/GTGCTTAAAATCTTTATAAGAGTAATGCGATAAAACGATAAGATTGCAGCATTACACG
*hmsS*	CGATACCGTTGAGGATTTATATTCTCAGCGGTTTGACGACAGATTGCAGCATTACACG/TATCCAGAACTTTTACGGATTCATAATAGAGTGGCGCGCTTGTAACGCACTGAGAAGC
Complementation of the *fur* mutant	
*fur*	AGACCGCCAACCTGAACTG/CAACGAAGAATAGCCACCTGAC
Protein expression	
*fur*	GCGGGATCCATGACTGACAACAACAAAG/GCGAAGCTTTTATCTTTTACTGTGTGCAGA
Primer extension	
*hmsH*	/TATTGTTGCAAAGTCATTATAGGAT
*hmsT*	/GGTATTTATTCCGACATCACGAC
YPO0450	/AGTAGCGGTAGTCATTTTTACG
LacZ reporter fusion	
*hmsH*	GCGGGATCCACTTTGCTGAAGACTTGTCACG/GCGAAGCTTCCGCCATAGCAGGATTAACG
*hmsT*	GCGGAATTCGCCCAGTACAGGTAACAAGG/GCGGGATCCCTGATCGTAGGAGTGGCTATTC
YPO0450	TCTGGATCCCTTACTGGTTGCTATTGCC/TCTAAGCTTGAGGTTCATGATGTTCATCA
EMSA	
*hmsH*	ACTTTGCTGAAGACTTGTCACG/CCGCCATAGCAGGATTAACG
*hmsT*	GCCCAGTACAGGTAACAAGG/CTGATCGTAGGAGTGGCTATTC
YPO0450	CTTACTGGTTGCTATTGCC/GAGGTTCATGATGTTCATCA
DNase I footprinting	
*hmsT*	GCCCAGTACAGGTAACAAGG/TTTGTTTCAGCCTGTCATCATG
	CATGATGACAGGCTGAAACAAA/CTGATCGTAGGAGTGGCTATTC

A PCR-generated DNA fragment containing the *fur* coding region together with its promoter-proximal region (458 bp upstream the coding sequence) and transcriptional terminator (189 bp downstream) was cloned into the pACYC184 vector (GenBank accession number X06403) that harbors a chloramphenicol resistance gene. Upon being verified by DNA sequencing, the recombinant plasmid was introduced into *Δfur*, yielding the complemented mutant strain *C-fur*.

The incubation temperature of 26°C was employed for the *Y. pestis* cultivation, unless otherwise specifically indicated. For the general bacterial cultivation and maintenance, *Y. pestis* was cultivated in the Luria-Bertani (LB) broth or on the LB agar plate. For preparing the glycerol stocks of bacterial cells, a single colony was inoculated on the LB agar plate for further incubation for 1 to 2 d; the bacterial cells were washed into the LB broth at an optical density at 620 nm (OD_620_ ) of about 1.5, and stored with addition of 30% glycerol at −80°C. For primer extension or LacZ fusion, 200 µl of bacterial glycerol stocks were inoculated into 18 ml of fresh LB broth, and allowed to grow with shaking at 230 rpm to an OD_620_ of 0.4 to 0.5 prior to the bacterial harvest.

### RNA Isolation and Primer Extension Assay

Before bacterial harvest, double-volume RNAprotect Bacteria Reagent (Qiagen) was added immediately to each cell culture. Total bacterial RNAs were extracted using the TRIzol Reagent (Invitrogen) [17]. RNA quality was monitored by agarose gel electrophoresis, and RNA quantity was determined by spectrophotometry. For the primer extension assay [17], an oligonucleotide primer complementary to a portion of the RNA transcript of each indicated gene was employed to synthesize cDNAs from the RNA templates. One to 10 µg of total RNA from each strain was annealed with 1 pmol of [γ-^32^P] end-labeled reverse primer using a Primer Extension System (Promega) according to the manufacturer’s instructions. The same labeled primer was also used for sequencing with the fmol® DNA Cycle Sequencing System (Promega). The primer extension products and sequencing materials were concentrated and analyzed in a 6% polyacrylamide/8 M urea gel. The result was detected by autoradiography (Kodak film).

### LacZ Reporter Fusion and β-Galactosidase Assay

The promoter-proximal DNA region of each gene tested was prepared by PCR with the Takara ExTaq DNA polymerase by using *Y. pestis* 201 genome DNA as template, and then cloned directionally into the *Hind*III*-Bam*HI site of the transcriptional fusion vector pRW50 [18] that contained a promotorless *lacZ* reporter gene. Correct clone was verified by DNA sequencing. Each *Y. pestis* strain tested was transformed with the recombinant plasmids. The empty plasmid was also introduced into each strain as negative control. The β-Galactosidase activity was measured on cellular extracts from cells cultivated as above by using the β-Galactosidase Enzyme Assay System (Promega) [17].

### Preparation of 6×His-tagged Fur (His-Fur) Protein

To prepare a His-Fur protein [14], the entire coding region of *fur* was amplified from *Y. pestis* 201 and cloned directionally into the *Bam*HI and *Hind*III site of plasmid pET28a (Novagen), which was verified by DNA sequencing. The recombinant plasmids encoding the His-Fur protein were transformed into *Escherichia coli* BL21 (DE3) cells (Novagen). Expression of His-Fur protein was induced by addition of 1 mM isopropyl-beta-D-thiogalactoside. The His-Fur protein was purified under native conditions with a QIAexpressionist™ Ni-NTA affinity chromatography (Qiagen). The purified, eluted protein was concentrated with the Amicon Ultra-15 (Millipore) to a final concentration of about 0.1 to 0.3 mg/ml in the storage buffer (PBS, pH 7.5 plus 20% glycerol). The protein purity was verified by SDS-PAGE with silver staining. The purified protein was stored at −80°C.

### Electrophoretic Mobility Shift Assay (EMSA)

For EMSA [14], promoter-proximal DNA regions were prepared by PCR amplification for EMSA. EMSA was performed using the Gel Shift Assay Systems (Promega). The 5′ ends of DNA were labeled using [γ-^32^P] ATP and T4 polynucleotide kinase. DNA binding was performed in a 10 µl volume containing binding buffer [100 µM MnCl_2_, 1 mM MgCl_2_, 0.5 mM DTT, 50 mM KCl, 10 mM Tris-HCl (pH 7.5), 0.05 mg/ml sheared salmon sperm DNA, 0.05 mg/ml BSA and 4% glycerol], labeled DNA (1000 to 2000 c.p.m/µl) and increasing amounts of His-Fur. We still included two control reactions: one contained the specific DNA competitor (unlabeled promoter DNA regions; cold probe), while the other was the non-specific protein competitor (rabbit anti-F1-protein polyclonal IgG antibody). After incubation at room temperature for 30 min, the products were loaded onto a native 4% (w/v) polyacrylamide gel and electrophoresed in 0.5×TB buffer containing 100 µM MnCl_2_ for 30 min at 220 V. Radioactive species were detected by autoradiography.

### DNase I Footprinting

For DNase I footprinting [14], promoter-proximal DNA regions were prepared by PCR amplification performed with specific primer pairs including a 5′-^32^P-labeled forward or reverse one and its non-labeled counterpart. The PCR products were purified using Qiaquick columns (Qiagen). Increasing amount of purified His-protein was incubated with the labeled DNA fragment (2 to 5 pmol) for 30 min at room temperature in a final volume of 10 µl containing binding buffer same as EMSA. Before DNA digestion, 10 µl of Ca^2+^/Mg^2+^ solution (5 mM CaCl_2_ and 10 mM MgCl_2_) was added, followed by incubation for 1 min at room temperature. Then, the optimized RQ1 RNase-Free DNase I (Promega) was added to the reaction mixture, and the mixture was incubated at room temperature for 30 to 90 s. The cleavage reaction was stopped by adding 9 µl of the stop solution (200 mM NaCl, 30 mM EDTA and 1% SDS) followed by DNA extraction and precipitation. The partially digested DNA samples were then analyzed in a 6% polyacrylamide/8 M urea gel. Protected regions were identified by comparison with the sequence ladders. For sequencing, the fmol® DNA Cycle Sequencing System (Promega) was used. The result was detected by autoradiography (Kodak film).

### Crystal Violet (CV) Staining of Biofilms

Two-hundred microlitre of bacterial glycerol stocks were spotted on the LB agar plate for further incubation for 1 to 2 d. The resulting bacterial cells were washed into the LB broth with an OD_620_ value of at least 1.0, stored at 4°C for cold shock for 8 to 12 h, and then diluted to an OD_620_ value of 0.8 with fresh LB broth. The diluted cultures were transferred into the 24-well tissue culture plates with 1 ml of cultures in each well, and allowed to grow at 230 rpm for 24 h. The media containing the planktonic cells were removed for determining the OD_620_ values. The well with the adherent biofilms was gently washed three times with 2 ml of H_2_O, and then incubated at 80°C for 15 min for the fixation of attached cells. The surface-attached cells were stained with 2 ml of 0.1% crystal violet for 15 min. The solution was removed, and the well was washed three times with 2 ml of H_2_O. Bound dye in the well was dissolved with 3 ml of dimethylsulfoxide. The OD_570_ values were recorded to indicate the crystal violet staining. The OD_570_/OD_620_ values were calculated to indicate the relative biofilm formation. The OD_620_ values were used for normalization to avoid the effect of growth rate and cell density.

### *Caenorhabditis Elegans* Biofilm Assays

The lawns of biofilm-negative *Escherichia coli* OP50, a uracil auxotroph whose growth was limited on the NGM (Nematode Growth Medium) agar plates, were used as the standard foods for *C. elegans*. When the larvae or adults of *C. elegans* grow on the lawns of *Y. pestis*, this bacterium creates biofilms to cover primarily on the nematode head by blanketing the mouth and thus inhibiting the nematode feeding, which has been developed as a model for *Yersinia* biofilm research [19], [20]. Bacterial strains were transformed with the pBC-GFP vector [21] to generate *Y. pestis* WT-GFP, *Δfur*-GFP, *ΔhmsS*-GFP, and *E. coli* OP50-GFP, respectively. To make the bacterial lawns, 200 µl of bacterial glycerol stocks were spotted on the LB agar plate for further incubation for 1 to 2 d. The resulting bacterial cells were washed into the LB broth with an OD_620_ value of at least 1.5, and aliquots of 300 µl were spotted on the LB agar plate for further incubation for 24 h. About 30 nematodes (adults or L4-stage larvae) were placed on each bacterial lawn expressing GFP, followed by incubation at 20°C for 12 h. The nematodes were suspended in the sterile M9 buffer (4.2 mM Na_2_HPO_4_, 2.2 mM KH_2_PO_4_, 8.55 mM NaCl, and 1 mM MgSO_4_) and then washed twice with M9 to remove planktonic bacteria. Worms was examined immediately by the epifluorescence microscopy.

### Colony Morphology Assay

Aliquots of 5 µl of bacterial glycerol stocks were spotted on the LB plate, followed by the incubation for one week. The photograph of surface morphology of each bacterial colony was recorded.

### Determination of Intracellular Levels of c-di-GMP

The intracellular levels of c-di-GMP were determined by a chromatography-coupled tandem mass spectrometry (HPLC-MS/MS) method as described previously [22]. Two-hundred microlitre of bacterial glycerol stocks were spotted on the LB agar plate for further incubation for 1 to 2 d. The resulting bacterial cells were washed into the LB broth with an OD_620_ value of about 0.5, and then aliquots of 5 ml were harvested for the extraction of c-di-GMP with the extraction solvent (acetonitrile: methanol:water = 40∶40∶20, v/v/v). The chromatographic separation was performed on a Spark HPLC system equipped with a binary pump system and a 200 µl sample loop. The analyte detection was performed on an API 4000-QTRAP quadrupole mass spectrometer equipped with an electro spray ionization source (Applied Biosystems).The serially diluted water solutions of HPLC-grade c-di-GMP (KeraFAST) were used for determining the standard curves. The HPLC-grade xanthosine 3′,5′-cyclic monophosphate (c-XMP, Sigma) was added as the internal standard into the c-di-GMP extract or standard solution at a final concentration of 50 ng/ml. Aliquots of 1 ml of bacterial cultures were harvest, and the amount of whole-cell protein was determined with a Micro BCA Protein Assay Kit (Thermo Scientific). The final c-di-GMP concentrations were expressed as pmol/mg of bacterial protein.

### Experimental Replicates and Statistical Methods

For phenotypic assays and LacZ fusion, experiments were performed with at least three independent bacterial cultures, and the values were expressed as mean ± standard deviation. Paired Student’s *t-*test was performed to determine statistically significant differences, and *P*<0.01 was considered to indicate statistical significance. For primer extension, EMSA, and DNase I footprinting, the representative data from at least two independent biological replicates were shown.

### Computational Promoter Analysis

The 300 bp upstream regions of the genes tested (Table 2) were retrieved with the ‘*retrieve-seq*’ program [23]. The PSSM [13] representing the conserved signals for Fur recognition in *Y. pestis* was used for the pattern matching within the target upstream DNA regions, by using the *matrices-paster* tool [23].

**Table 2:** Computational promoter analysis.

Operon	First gene	Patten matching		
		Fur box-like sequence	Position ^δ^	Sore
*hmsT*	*hmsT*	AATGATAATCATAACCAAT	D-272…−254	15.07
		AACAATAATAATTCCCAAC	D-95…−77	8.74
*hmsHFRS*	*hmsH*	AATGATGATGAAATGGAAT	R-94…−76	4.58
YPO0450-0448	YPO0450	AATAAGATTTAAGATAAAT	D-139…−121	3.89

A PSSM [13] representing the conserved signals for Fur recognition in *Y. pestis* was used for the prediction of Fur-box like sequences within the 300 bp upstream DNA regions of the major biofilm-required genetic loci *hmsT, hmsHFRS*, and YPO0450-0498. The diguanylate cyclase gene YPO0449 was located in the putative operon YPO0450-0498. δ, the minus numbers indicated the nucleotide positions upstream of translation start, and D and R represented the direct and reverse sequences, respectively.

## Results

### Fur Inhibited Biofilm Formation

**Figure 1:**
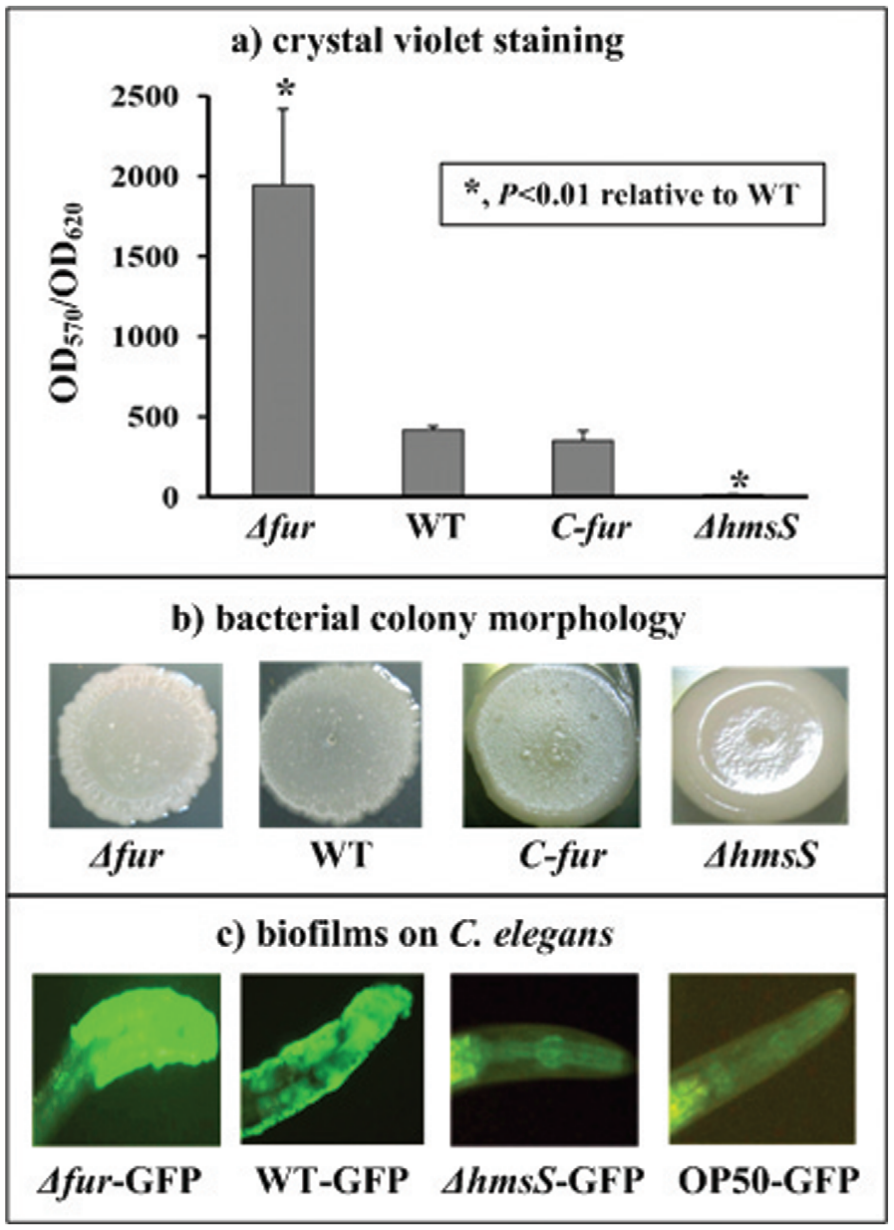
*Yersinia pestis* biofilms assays. a) Adherent bacterial biomass determined by crystal violet staining. *Y. pestis* was grown in the 24-well polystyrene dishes, and the biomass adherent to the well wall was stained with crystal violet to determine the OD_570_ values. The planktonic cells were subjective to determine the OD_620_ values (i.e., cell density) for normalization. Shown were the OD_570_/OD_620_ values representing the relative capacity of biofilm formation of each strain tested. **b) Bacterial colony morphology.** Aliquots of 5 µl of bacterial glycerol stocks were spotted on the LB plate, followed by the incubation for one week. **c)** ***Yersinia*** **biofilms on** ***C. elegans*** **.** The adult or L4 nematodes were spread on the lawn of *Y. pestis* expressing GFP and allowed to grow for 12 h. Shown were biofilms attach to the head posterior to the nematode mouth.

Growing in the polystyrene microtiter plate, *Y. pestis* cells tend to attach to the walls [9]. The attached biomass (i.e., *in vitro* biofilms) can be detected with CV staining, which has been developed long time ago as a model for the determination of *in vitro* biofilms [24]. Herein, *Δfur* gave the normalized CV staining significantly greater than WT that was comparable to the complemented mutant *C-fur*, while the biofilm-negative strain *ΔhmsS* gave almost no CV straining (Fig. 1a).

Biofilm-forming bacteria growing on the agar plate can give a rugose colony morphology in which the cells are embedded in abundant biofilm exopolysaccharide, and the degrees of rugose colony morphology positively reflect the ability to synthesize the biofilm exopolysaccharide [9], [25], [26]. *Δfur* produced colonies with much more rugose morphology in relative to WT that was comparable to *C-fur*, while *ΔhmsS* made the smooth colonies (Fig. 1b). These suggested that *Δfur* overproduced the biofilm exopolysaccharide relative to WT.

*Yersinia* biofilms adhere to the surface of *C. elegans*, primarily on the head to cover the mouth. When the adult or L4 nematodes were placed on the lawn of *Y. pestis* expressing GFP and allowed to grow for 12 h, *Δfur-*GFP produced more extensive and denser biofilms than WT*-*GFP, while no biofilm was detectable for *ΔhmsS-*GFP (negative control) and *E. coli* OP50 (blank control) (Fig. 1c).

Taken together, *Y. pestis* Fur acted as a repressor for the biofilm formation, most likely through inhibiting the production of biofilm exopolysaccharide.

### *hmsT* was Predicted to be a Direct Fur Target

The Fur PSSM [13] was used to statistically predict the presence of Fur box-like elements [14] within the promoter-proximal regions of the three major biofilm-required loci *hmsHFRS*, *hmsT*, and YPO0450-0448. This analysis generated a weight score for each target promoter, and the higher score value indicated the higher probability of the Fur-promoter association [14]. When a frequently used score of 7 was taken as the cutoff value, Fur box-like sequences were found for *hmsT* rather than the remaining two (Table 2). This computational promoter analysis suggested that Fur could recognize the *hmsT* promoter for transcriptional regulation.

### Fur Repressed *hmsT* Transcription in a Direct Manner

**Figure 2:**
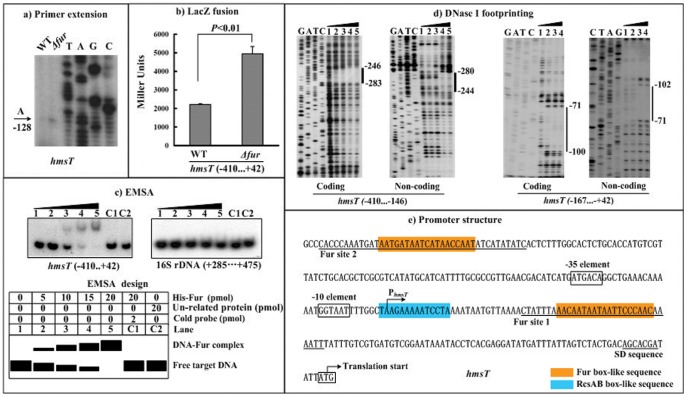
Repression of *hmsT* by Fur. The positive and minus numbers indicated the nucleotide positions upstream and downstream of the translation start, respectively. Lanes G, A, T and C represented the Sanger sequencing reactions. **a) Primer extension.** An oligonucleotide primer was designed to be complementary to the RNA transcript of *hmsT*. The primer extension products were analyzed with 8 M urea-6% acrylamide sequencing gel. Shown with the arrow was the transcription start of *hmsT*. **b) LacZ fusion.** A promoter-proximal region of *hmsT* was cloned into the *lacZ* transcriptional fusion vector pRW50, and transformed into WT or *Δfur* to determine the *hmsT* promoter activity, i.e., the β-Galactosidase activity (Miller units) in the cellular extracts. **c) EMSA.** The radioactively labeled promoter-proximal DNA fragment of *hmsT* was incubated with increasing amounts of purified His-Fur protein, and then subjected to 4% (w/v) polyacrylamide gel electrophoresis; with the increasing amounts of His-Fur, the band of free target DNA disappeared, and a retarded DNA band with decreased mobility turned up, which presumably represented the protein-DNA complex. A DNA fragment from the coding region of the 16S rRNA gene served as a negative control. **d) DNase I footprinting.** The labeled coding or non-coding DNA probes were incubated with various amounts of purified His-Fur (lanes 1, 2, 3 and 4, and 5 contained 0, 5, 10, 15 and 20 pmol, respectively), and subjected to DNase I footprinting assay. The protected regions (bold line) were indicated on the right-hand side. **e) Promoter structure.** Shown were translation/transcription starts, SD sequences, promoter −10 and −35 elements, Fur sites, and Fur/RcsAB box-like sequences for *hmsT*.

The primer extension experiments (Fig. 2a) were conducted to determine the yield of primer extension product of *hmsT* (i.e., the relative *hmsT* transcription level) in WT or *Δfur*. A single transcriptional start site was detected to be located at the nucleotide A that was 128 bp upstream of *hmsT*, and thus, a single promoter was transcribed for *hmsT*. The primer extension assay also disclosed that the mRNA level of *hmsT* considerably enhanced in *Δfur* relative to WT.

To test the action of Fur on the promoter activity of *hmsT*, we constructed an *hmsT::lacZ* fusion vector, containing a 453 bp promoter-proximal region of *hmsT* and the promoterless *lacZ*, which was then transformed into WT or *Δfur* (Fig. 2b). The β-galactosidase activity was measured for evaluating the *hmsT* promoter activity in each strain. The LacZ fusion experiments disclosed that the *hmsT* promoter activity significantly enhanced in *Δfur* relative to WT.

EMSA was conducted to answer whether Fur would bind to the *hmsT* upstream region *in vitro* (Fig. 2c). As expected, a purified His-Fur bound to the labeled *hmsT* promoter DNA in a dose-dependent manner. To confirm the specificity of Fur-DNA association, the EMSA experiments still included a partial coding region of the 16S rRNA gene, and the negative results were obtained.

In order to locate the precise Fur sites, DNase I footprinting experiments were performed with both coding and non-coding strands of target DNA fragments (Fig. 2d). Since two Fur box-like sequences were predicted for *hmsT*, two distinct *hmsT* promoter-proximal regions, containing the above predicted elements respectively, were subjected to the footprinting experiments. The results confirmed the binding of His-Fur to the two target DNA fragments *in vitro*. His-Fur protected a single region within each of the two target DNA fragments tested against DNase I digestion in a dose-dependent pattern. The two footprints were located from 283 to 244 bp (site 2) and from 102 to 71 bp (site 1) upstream of *hmsT*, respectively. Both of them contained the Fur box-like sequences, and were considered as the Fur sites for *hmsT* (Fig. 2e).

### Fur had no Regulatory Effect on *hmsHFRS* and YPO0450-0448

**Figure 3:**
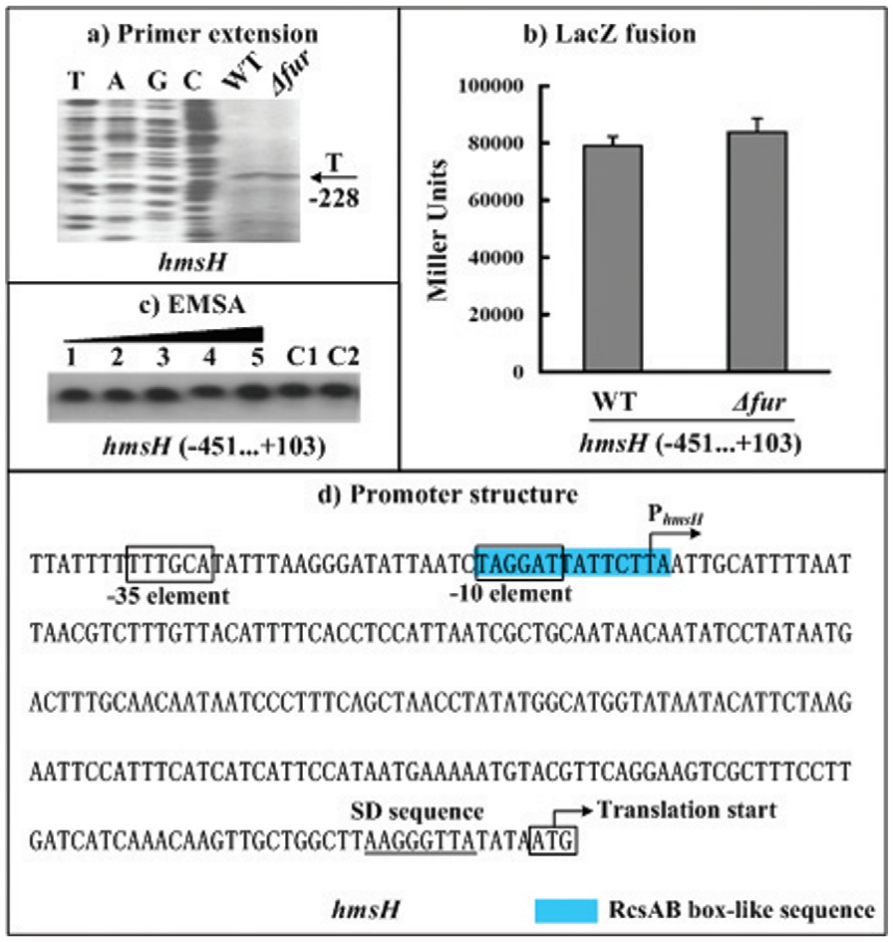
Fur had no regulatory action on *hmsH*. The positive and minus numbers of position indicated the nucleotide positions upstream and downstream of the translation start, respectively. **a) Primer extension.** An oligonucleotide primer was designed to be complementary to the RNA transcript of *hmsH*. The primer extension products were analyzed with 8 M urea-6% acrylamide sequencing gel. Lanes C, T, A, and G represented the Sanger sequencing reactions. Shown with the arrow was the transcription start of *hmsH*. **b) LacZ fusion.** A promoter-proximal region of *hmsH* was cloned into the *lacZ* transcriptional fusion vector pRW50, and transformed into WT or *Δfur* to determine the *hmsH* promoter activity (Miller units) in the cellular extracts. **e) Promoter structure.** Shown were translation/transcription starts, SD sequences, promoter −10 and −35 elements, and RcsAB box-like sequence for *hmsH*.

**Figure 4:**
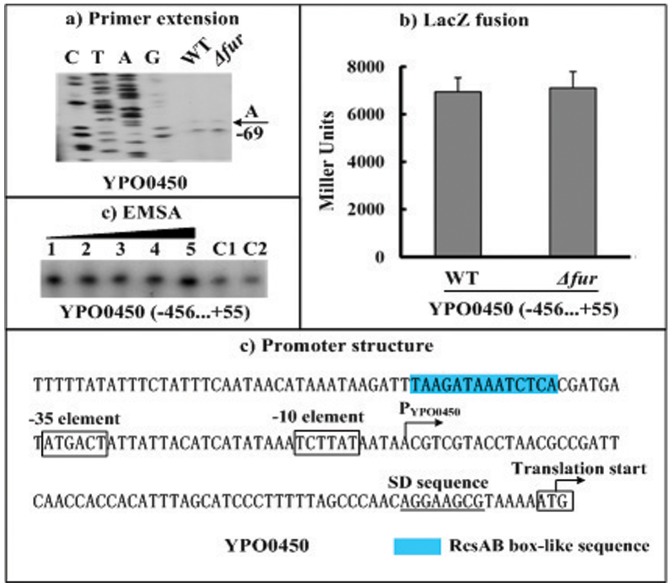
Fur had no regulatory action on YPO0450. The positive and minus numbers of position indicated the nucleotide positions upstream and downstream of the translation start, respectively. **a) Primer extension.** An oligonucleotide primer was designed to be complementary to the RNA transcript of YPO0450. The primer extension products were analyzed with 8 M urea-6% acrylamide sequencing gel. Lanes C, T, A, and G represented the Sanger sequencing reactions. Two closely neighboring extension products were detected. Only the longer product was chosen for identifying the transcription start site shown with the arrow, due to the facts that the shorter extension product might represent the premature stops resulted from the difficulty of polymerase in passing difficult nucleotide sites, and that the core promoter −35 element could not be predicted for the shorter extension product. **b) LacZ fusion.** A promoter-proximal region of YPO0450 was cloned into the *lacZ* transcriptional fusion vector pRW50, and transformed into WT or *Δfur* to determine the YPO0450 promoter activity (Miller units) in the cellular extracts. **e) Promoter structure.** Shown were translation/transcription starts, SD sequences, promoter −10 and −35 elements, and RcsAB box-like sequence for YPO0450.

The gene regulation experiments still included the first genes (*hmsH* and YPO0450) of the *hmsHFRS* and YPO0450-0448 operons. The primer extension (Fig. 3a and 4a) and LacZ fusion (Fig. 3b and 4b) assays were conducted for *hmsH* and YPO0450. It was revealed that the *fur* null mutation have no influence on the *hmsH*/YPO0450 transcription (Fig. 3a and 4a) or on the *hmsH*/YPO0450 promoter activity (Fig. 3b and 4b). In addition, the EMSA experiments (Fig. 3c and 4c) indicated that His-Fur could not bind to the upsteam DNA regions of *hmsH* and YPO0450. Therefore, the Fur regulator had no regulatory action on *hmsHFRS* and YPO0450-0448 at the transcriptional level under the growth conditions tested herein.

### Fur Repressed c-di-GMP Production

**Figure 5:**
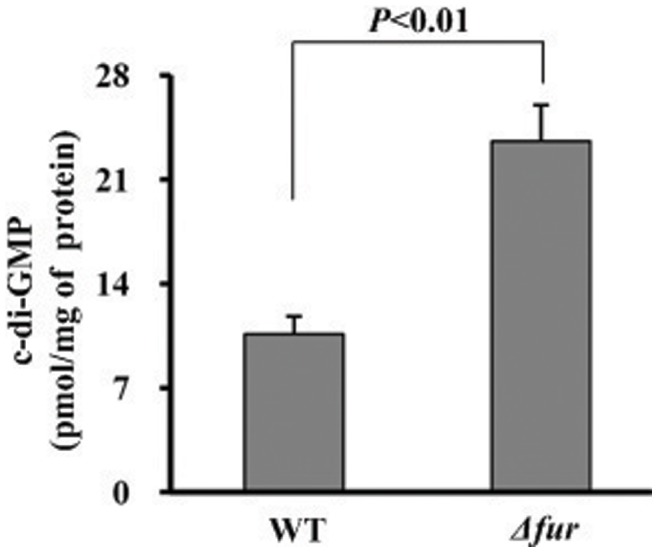
Production of c-di-GMP in different strains. The intracellular c-di-GMP concentrations were determined by a HPLC-MS/MS method, and the determining values were expressed as pmol/mg of bacterial protein (see supplementary Fig. S1 for representative HPLC-MS/MS traces).

The intracellular levels of c-di-GMP were determined in WT and *Δfur* by a HPL-MC/MS method. Compared to WT, a significantly enhanced production of c-di-GMP was observed for *Δfur* (Fig. 5). These results verified that the Fur-mediated transcriptional repression *hmsT* accounted for the inhibition of c-di-GMP synthesis by Fur in *Y. pestis*.

## Discussion

*Y. pestis* is a recently (from the evolutionary point of view) merged clone of the mild enteric pathogen *Y. pseudotuberculosis* [27]. *Y. pseudotuberculosis* is transmitted by the food-borne route, while *Y. pestis* utilizes a radically different mechanism of transmission that rely primarily upon bite of fleas [28]. All of the known structural genes required for the biofilm formation are harbored in *Y. pseudotuberculosis*, but typical *Y. pseudotuberculosis* cannot synthesize adhesive biofilms on nematodes and make blockage in fleas [29].

The *Y. pseudotuberculosis* NghA is a glycosyl hydrolase that cleaves the β-linked N-acetylglucosamine residues, and thus, it plays a key role in degrading the biofilm exopolysaccharide [30].

The RcsAB box-like sequence can be predicted within the promoter-proximal regions of *hmsT* (Fig. 2e), *hmsHFRS* (Fig. 3e), and YPO0450-0448 (Fig. 4e). Repression of the *hmsT* transcription by RcsAB through the RcsAB-promoter association has been established recently [31]. *hmsHFRS* and YPO0450-044 appears to be the additional direct RcsAB targets (unpublished data), and thus, RcsAB acts as a repressor of *Yersinia* biofilm formation through inhibiting the production of both c-di-GMP and biofilm exopolysaccharide.

Data presented here disclosed that the Fur regulator had a negative effect on the biofilm formation through repressing the *hmsT* transcription. DNase I footprinting experiments precisely determined the Fur sites for *hmsT*. The primer extension assays mapped a single promoter transcribed for *hmsT*, and accordingly, the core promoter −10 and −35 elements for RNA polymerase recognition were predicted. Collection of data on the translation/transcription start sites, Shine-Dalgarno (SD) sequence (a ribosomal binding site in the mRNA), core promoter −10 and −35 elements for RNA polymerase recognition, and two *cis*-acting sites for Fur recognition, enabled us to depict the organization of Fur-dependent promoter of *hmsT* herein (Fig. 2e).

The two Fur sites were located downstream and upstream of the transcription start site of *hmsT*, respectively, while the RcsAB box-like sequence overlapped the *hmsT* transcription start. The binding of Fur or RcsAB to the *hmsT* promoter regions would block the entry of the RNA polymerase to repress the *hmsT* transcription. In addition, no change in the transcription of *hmsHFRS* or YPO0450-0448 was observed in the *fur* mutant compared to its parent strain, indicating that Fur had no regulatory activity on *hmsHFRS* and YPO0450-0448.

Since the genomic regions encoding Fur, HmsT, HmsHFRS, and YPO0450-0448 were extremely conserved between *Y. pestis* and typical *Y. pseudotuberculosis* [32], the regulatory circuit determined herein could be applied to *Y. pseudotuberculosis*. The action of at least three anti-biofilm factors NghA, RcsAB, and Fur will bring a tight biofilm-negative phenotype of typical *Y. pseudotuberculosis.* In contrast, *Y. pestis* has undergone the evolution of loss-of-function of NghA [33] and RcsA [9], which will confer a selective advantage to the progenitor *Y. pestis*. The mutational loss of function of Fur is of virtual impossibility, since Fur is a predominant regulator of iron assimilation in *Y. pestis* [13], [14]. Fur-mediated repression of *hmsT* expression and c-di-GMP synthesis would greatly contribute to finely modulate *Yesinia* biofilm production within the physiological range. Moreover, *Y. pestis* has acquired an additional factor Ymt that promotes the bacterial survival of in fleas [30]. The above evolutionary events make *Y. pestis* prerequisitely survive in fleas and moreover synthesize adhesive biofilms in flea proventriculus to make the blockage, resulting in an efficient arthropod-borne transmission [34].

## Supporting Information

Figure S1 **Representative HPLC-MS/MS traces.** c-di-GMP in a extract of WT (a) or *Δfur* (b), and a standard sample of c-di-GMP (c) in water at a concentration of 0.3 nM were detected by HPLC-MS/MS -di-GMP c-di-GMP. cXMP was used as the internal standard at a concentration of 50 ng/ml.
(JPG)
