# Supplementary figures and images for: Fur Is a Repressor of Biofilm Formation in Yersinia pestis
Source: PLoS One. 2012 Dec 21;7(12):e52392. doi: 10.1371/journal.pone.0052392 (PMC3528687; doi:10.1371/journal.pone.0052392)

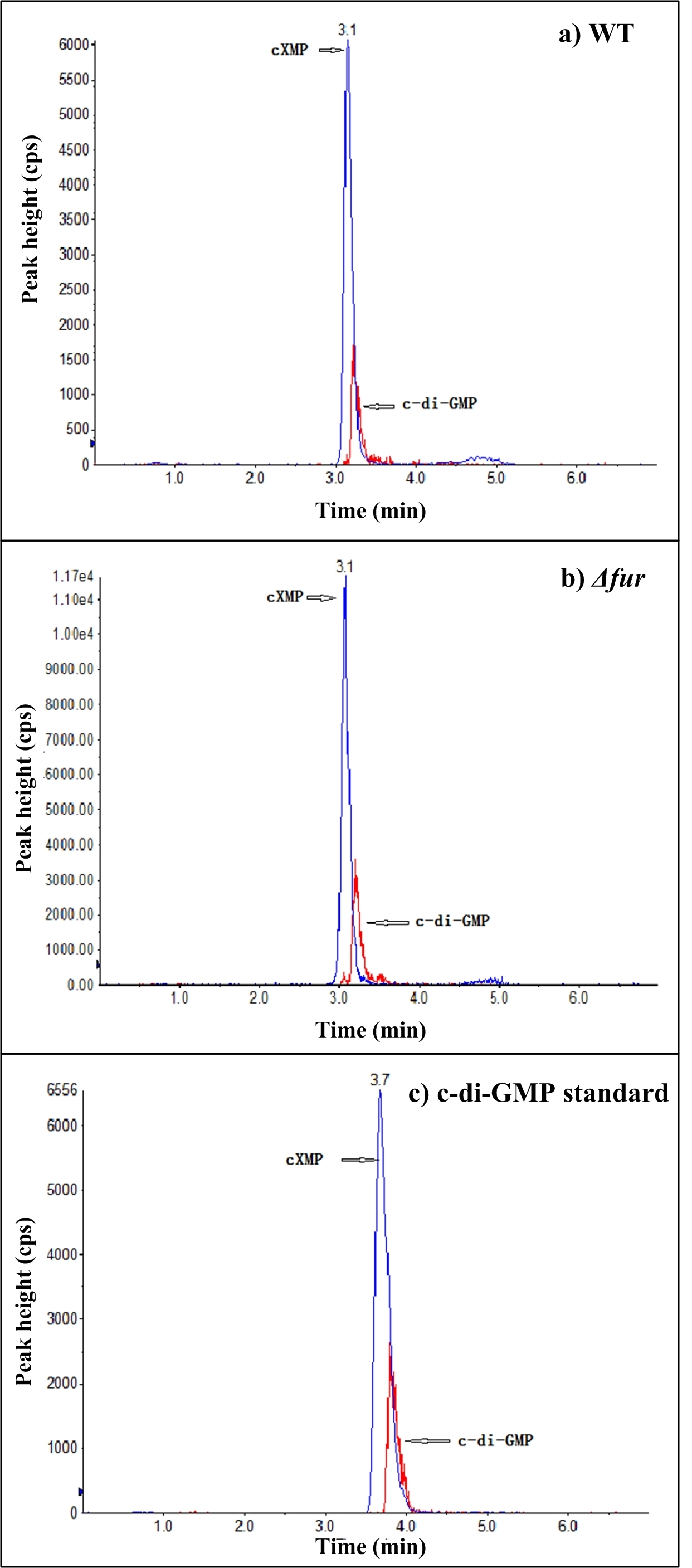

Supplement: Figure S1 — Representative HPLC-MS/MS traces. c-di-GMP in a extract of WT (a) or Δfur (b), and a standard sample of c-di-GMP (c) in water at a concentration of 0.3 nM were detected by HPLC-MS/MS -di-GMP c-di-GMP. cXMP was used as the internal standard at a concentration of 50 ng/ml. (JPG) [file pone.0052392.s001.jpg]
